# Community-based intervention for blood pressure reduction in Nepal (COBIN trial): study protocol for a cluster-randomized controlled trial

**DOI:** 10.1186/s13063-016-1412-3

**Published:** 2016-06-18

**Authors:** Dinesh Neupane, Craig S. McLachlan, Bo Christensen, Arjun Karki, Henry B. Perry, Per Kallestrup

**Affiliations:** Center for Global Health, Department of Public Health, Aarhus University, Bartholins Allé 2, 8000 Aarhus C, Denmark; Rural Clinical School, University of New South Wales, Samuels Building, Sydney, NSW 2052 Australia; Department of Public Health, Institute of General Medical Practice, Aarhus University, Aarhus, Denmark; Patan Academy of Health Sciences, Kathmandu, Nepal; Department of International Health, Health Systems Program, Bloomberg School of Public Health, The Johns Hopkins University, Baltimore, MD USA

**Keywords:** Female Community Health Volunteers, Primary Health Care, Non-communicable diseases, Hypertension, Community-based, Community health worker, Nepal

## Abstract

**Background:**

Hypertension contributes to a significant burden of cardiovascular disease in low- and middle-income countries; however, responses are inadequate because of a lack of conclusive evidence on population-based approaches to hypertension control.

**Methods/design:**

The objective of the present study is to determine the effect of family-based home health education and blood pressure monitoring by trained female community health volunteers. The primary outcome is change in mean systolic blood pressure. A community-based, open-masked, two-armed, cluster-randomized trial will be conducted in Lekhnath Municipality of Nepal. The municipality is divided into 15 administrative clusters. Randomization will be conducted for 14 clusters: 7 for the intervention arm and 7 for the control arm. The participants were recruited from a prevalence study conducted earlier. On the basis of population proportion size, 929 individuals for the intervention group and 709 individuals for the control group will participate in the study. Due to the nature of the study, study participants are not compensated or insured. As part of the blood pressure intervention, trained female community health volunteers will conduct home visits for health education and blood pressure measurement. The primary outcomes will be modeled by using multiple linear regression analysis.

**Discussion:**

This project will be an investigation of a community-based intervention to control blood pressure in countries with limited resources. The study will provide detailed information on the burden of blood pressure and also whether treatment targets are being met. Moreover, evidence will be provided on the future role of female community health volunteers for hypertension management in Nepal. The lessons learned from this study may also be replicated in other rural areas of Nepal and elsewhere in the world with similar settings.

**Trial registration:**

ClinicalTrials.gov NCT02428075. Registered on 23 April 2015.

**Electronic supplementary material:**

The online version of this article (doi:10.1186/s13063-016-1412-3) contains supplementary material, which is available to authorized users.

## Background

The burden of hypertension worldwide contributes significantly to heart failure, coronary artery events, stroke, kidney failure, disability, and premature death [1]. Modifiable lifestyle behaviors such as tobacco use, physical inactivity, unhealthy diet, and alcohol abuse are the major risk factors contributing to the rising incidence of high blood pressure [2]. A population-based approach to decreasing blood pressure levels in the general population by even modest levels has the potential to substantially reduce morbidity and mortality and possibly to delay the onset of hypertension [3]. It has been estimated that a 5-mmHg reduction of systolic blood pressure (SBP) in the population would result in a 14 % overall reduction in mortality due to stroke, a 9 % reduction in mortality due to coronary heart disease, and a 7 % decrease in all-cause mortality [3, 4]. A study published in 1998 showed that the association between blood pressure and stroke in East Asia seems higher than in Western populations. A population-wide reduction of 3 mmHg in diastolic blood pressure is predicted to eventually decrease the number of strokes by about one-third [5].

**Fig. 1:**
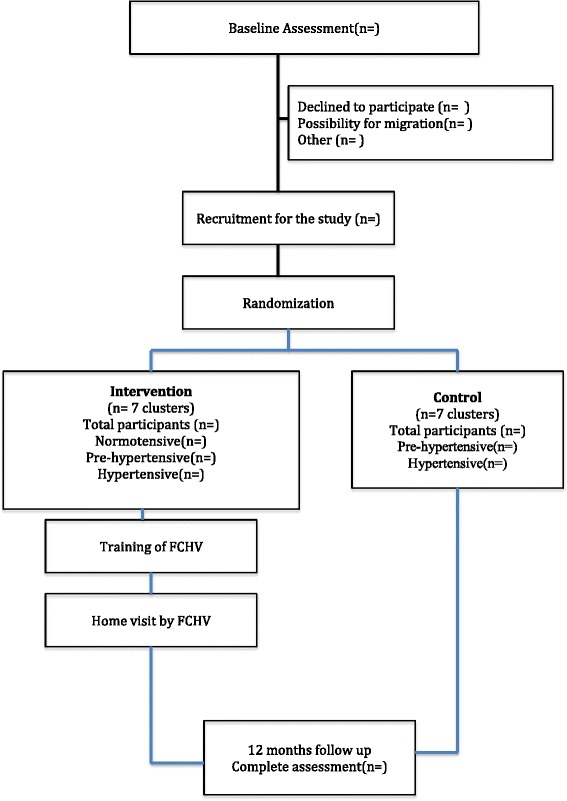
Planned flow of participants through the trial. *FCHV* female community health volunteer

However, community-based protocols to reduce blood pressure via lifestyle interventions remain poorly defined. Two studies on health promotion for blood pressure reduction have yielded inconclusive results [6, 7]. We have identified deficiencies in study design in a number of previous studies, such as contamination bias. For example, there has been a lack of detail about health workers, and many were demonstration projects without a concomitant control group [8–13]. Reduction of dietary salt in Sub-Saharan Africa lowered blood pressure in the short term [14], and a study conducted in Pakistan showed small but significant differences in blood pressure among children and young adults in the intervention group compared with the control group [15]. However, there is still a lack of evidence on effective protocols for community health worker population approaches to reducing blood pressure in normotensive, prehypertensive, and hypertensive groups. Indeed, we noted in our previous meta-analysis that there is a variable prevalence of hypertension across South Asian Association for Regional Cooperation countries, with people in a number of countries having blood pressure above the global average. In Nepal, the prevalence of hypertension ranges from 22 % to 34 % [16]. We also noted that studies are not consistent in their data collection methods regarding hypertension and related modifiable risk factors [16]. Hence, a benefit of the present study will be to provide an accurate prevalence rate and risk factors for hypertension, and also to design an intervention for hypertension management at the community level.

One of the possibilities for addressing emerging hypertension in low- and middle-income countries could be by involving community health workers, such as female community health volunteers (FCHVs) in the setting of Nepal [17]. In Nepal, there are over 50,000 FCHVs working to improve the health of their communities [18], and they are highly recognized for the contribution they have made to improving maternal and child health [19]. Nepal’s Ministry of Health and Population relies on FCHVs to act as health resources in their villages by educating their neighbors about important health issues and providing basic health services in the absence of other biomedical resources.

FCHVs are selected by members of Mothers’ Group for Health with the help of local health facility staff. They receive 18 days of basic training in two phases (9 + 9 days) on selected primary health care components. The current role of the FCHV is to promote health and healthy behaviors of community-dwelling people to promote safe motherhood, child health, and family planning [18]. According to the National Female Community Health Volunteer Survey, the median age of FCHVs is 37 years, and 42 % have completed primary school education or higher [20]. Despite FCHVs’ positive record of collaborating with the official health system, these cadres have yet to be mobilized for prevention, control, and treatment of hypertension. The initial service to be promoted in the present trial design is for the FCHV to be skilled in monitoring blood pressure, providing health promotion counseling, and referring at-risk patients to the nearest health center for hypertension treatment. In our earlier feasibility study, FCHVs expressed willingness and readiness to be trained in hypertension management [21]. The FCHVs serve as a unique link between the health facilities and local communities, and they have the potential to play an important role in promoting knowledge and awareness of healthier lifestyles and thereby reducing the risk of hypertension. Thus, the aim of this study protocol is to determine the effect of family-based home health education and screening of blood pressure in adults by FCHVs in Nepal.

## Methods/design

### Study design and setting

A community-based, open-masked, two-armed, cluster-randomized trial will be conducted in Lekhnath Municipality of Nepal. The municipality is located in the western region of Nepal, about 180 km west of Kathmandu. The total population of the municipality was 58,816 in the census carried out in 2011. The municipality is divided into 15 smaller units called *wards*. The municipality is a semiurban area with limited health services comprising one primary health care center, three sub-health posts, and two urban health care centers. According to information provided by District Public Health Office, Kaski in 2013, there were 123 FCHVs in the municipality. No community-based interventions for hypertension at the population level have been carried out in this region to date.

### Recruitment

A population framework of all eligible participants will be prepared from the voter list prepared by the Election Commission in 2007. As of 15 December 2006, all the people included in the voter list were at least 18 years old. By 2015, all the people in the voter list were at least 25 years of age. The voter list also contains information about the household. Using the voter list, a survey will be carried out by trained health professionals such as nurses and health assistants to estimate the proportion of hypertension in the municipality as well as to prepare a list of eligible respondents to participate in our randomized controlled trial. The data enumerators will receive 5 days of intensive training before collecting data.

If there is more than one participant from the same household eligible to participate in the study at the time of data collection, the Kish method will be adopted to select the participant [22]. The survey tool will be adapted from the World Health Organization (WHO) STEPwise approach to surveillance [23], which includes physical measurement (height, weight), sociodemographic information (e.g., age, sex, family size, occupation, income, education), lifestyle factors (e.g., salt consumption, smoking, alcohol, physical activity), and blood pressure measurement. The classification of normotension, prehypertension, and hypertension is shown in Table 1.

**Table 1:** Classification of normotension, prehypertension, and hypertension

SBP/DBP level, mmHg	Taking antihypertensive medication	Status for recruitment
< 120/80		
Yes	No	Normotensive
No	Yes	Hypertensive
≥ 120/80 and < 140/90		
Yes	No	Prehypertensive
No	Yes	Hypertensive
≥ 140/90		
Yes	No	Hypertensive
No	Yes	Hypertensive

*DBP* diastolic blood pressure, *SBP* systolic blood pressure

During that survey, normotensive, prehypertensive, and hypertensive participants will be identified and asked to participate in the study. From among eligible participants, the required sample size of respondents will be chosen in proportion to the population of the specific ward to be sampled. On the fifth day of training, FCHVs from one cluster will sit together and allocate the number of households that has to be visited on the basis of the baseline survey. After that, FCHVs will visit each household, meet the selected participant, and ask for consent to participate in the trial.

### Randomization

Of 15 wards (clusters), 1 ward will be excluded by lottery method before randomization. Clusters will be randomly assigned to intervention and control areas using a 1:1 allocation ratio. Randomization will be done before the participants are recruited. Research teams responsible for identifying potential participants, obtaining consent, and recruiting trial participants will be blinded to the participants’ allocation status. The following steps will be taken to avoid selection bias during random allocation: (1) clusters will be randomized only after the baseline survey; (2) clusters will not be withdrawn from or added to the study; and (3) an epidemiologist who does not come in contact with the study will randomize the clusters.

### Inclusion and exclusion criteria

People 25–65 years of age who are listed in the voter list of 2007 are eligible for inclusion in the baseline survey. The primary endpoint of the study is a reduction in SBP, because with aging there is an increase in SBP due to aortic stiffening [24]. This may not be modifiable through reduction in risk factors. Hence, we have defined an adult population within an age range who will likely respond to reductions in hypertension risk factors through educational interventions. Participants for the blood pressure education intervention study will be chosen from among the respondents who participated in the baseline survey and who live in a ward in the study area. Persons who are severely ill, who are unlikely to be in the community throughout the intervention, who are pregnant women, and who decline consent will be excluded from the study.

### Primary and secondary outcomes

The primary outcome of the study is a reduction in mean SBP among normotensive, prehypertensive, and hypertensive populations. The secondary outcomes of the study are reductions in mean diastolic blood pressure and proportion of risk factors (smoking, alcohol, salt intake, body mass index).

### Sample size calculation 

The sample size for the population prevalence study is estimated according to the method suggested in the WHO STEPwise approach [23]. We calculated the sample size for 95 % CI (*z* = 1.96) on the basis of a 5 % margin of error (α), a 25 % estimated prevalence of hypertension based on the latest nationwide study in Nepal [25], a design effect of 1, and a response rate of 80 %. We will include only an age group ≥ 25 years old, resulting in a total of four age groups for each sex (total strata = 8). This will result in a sample size equal to 2882. The participants in the baseline survey will be eligible to participate in the intervention study. For the intervention study, we took the reference of the mean difference and SD from studies conducted in Vietnam and Taiwan [26, 27]. As our project is randomized and includes visits by community health workers who measure blood pressure for screening additionally, we are hopeful that we will observe a 5-mmHg difference in mean SBP between the control and intervention arms. The detailed assumptions of the sample size calculation are presented in Table 2.

**Table 2:** Sample size calculation

Category	Mean difference (mmHg)	SD	Intracluster correlation coefficient	Design effect	Clusters per arm	Individuals per arm	Total sample size
Normotensive	5	18.8	0.01	1.57	7	406	812
Prehypertensive	5	13.6	0.01	1.23	7	168	336
Hypertensive	5	15.7	0.01	1.34	7	245	490

### Intervention

After randomization, the FCHVs participating in the intervention wards will receive a 5-day training package featuring (1) introduction and importance of noncommunicable disease and/or hypertension; (2) identifying populations at risk using a checklist comprising level of salt intake, physical inactivity, smoking, and alcohol consumption; (3) blood pressure screening by using digital sphygmomanometers, height and weight measurement, and referral for those whose blood pressure is > 140/90 mmHg; (4) providing health education in major risk factors; and (5) recording, reporting, and follow-up. Educational sessions are guided by use of the Health Belief Model [28], and training materials will be developed by involving local major stakeholders. The developed materials will be pretested with the FCHVs of Pokhara Municipality.

After receiving the training, the FCHVs will be given a digital sphygmomanometer, manual weighing scale, clinical measuring tape for height, an FCHV manual, and a recording register. During the training program, FCHVs will allocate the households themselves. On average, 1 FCHV will visit 25 households (13 normotensive, 5 prehypertensive, and 7 hypertensive). Each FCHV will visit selected households three times during 1 year, where the FCHV will provide counseling and measure the blood pressure as well as the height and weight of selected respondents. If the participant is hypertensive, the FCHV will ask the respondent to visit the health facility for further diagnosis and treatment. For hypertensive participants, the FCHV will also monitor whether the respondent is taking medicine as per the advice of health care workers. Participants in the control clusters will receive usual care. Usual care means current practices related to hypertension management at the community level. After the 1-year period of the intervention, a follow-up survey will be carried out through trained professional health workers such as nurses and health assistants.

### Minimization of contamination

The risk and level of contamination will be monitored at the levels of FCHVs and participants. FCHVs will be instructed not to share information about the study and not to provide any support to people from other clusters in the community, other than the ones who are assigned. The risk of contamination will also be minimized by the cluster design, whereby the intervention and control clusters will be geographically separated and the chance of intervention cluster participants regularly meeting control cluster participants will be negligible. Further, we will collect all possible means of contamination between intervention and control participants during the follow-up survey, and, if these are found to be significant, we will make adjustments while estimating the effect.

### Intervention fidelity

Intervention fidelity refers to the extent to which core components of interventions are delivered as intended in the protocols. Ensuring and measuring fidelity are important in detecting the effects of the intervention [29]. In the present study, a number of steps will be implemented to facilitate fidelity. The importance of fidelity will be explained to FCHVs of intervention clusters during the training session. FCHVs will also receive a register to remind them of the stages of the intervention and to assist the research team to monitor its delivery. Every 4 months, they will report to the research team the activities they have carried out.

### Data analysis

The quantitative analysis will be done using STATA version 14 software (StataCorp, College Station, TX, USA) following the Consolidated Standards of Reporting Trials (CONSORT) guidelines for cluster-randomized trials. Flowcharts (Fig. 1) will include the number of participants seen at each stage of the trial, including the number screened, eligible, randomized, and analyzed for the primary outcome. In initial analysis, we will compare baseline characteristics of enrolled participants in the study intervention arm and follow-up status. The primary analyses will be intention to treat at the 12-month follow-up, adjusted for the baseline measure of the outcome. In the event that randomization does not control for differences between the treatment and control groups on baseline characteristics, we will statistically control for those differences in subsequent analyses of program effects. The final outcome will be modeled by using random - effects regression models. The effect size will be reported as mean differences with 95 % CIs for primary data.

### Interim analyses and stopping rules

We do not have a plan for interim analysis, as we do not expect a situation that would lead us to stop the study. However, in the event of an extreme situation such as a natural disaster, a conflict, or duplication of similar interventions in our control area, we will temporarily stop the study to assess the implications on the study design.

### Trial management

We have a robust mechanism to ensure data quality. First, the data enumerator will receive intensive training on the process of data collection. The principal investigator will monitor the data enumerator on a day-to-day basis. The data entry clerk will inspect the data manually at first and enter the data in an EpiData software file (EpiData Association, Odense, Denmark). Ten percent of the data will be double-entered to qualify potential errors in data entry quality. The research support officer will cross-check the data quality on the spot and in our field office for any incomplete, inconsistent, and invalid data. Any deviation in the data quality will require retesting in the field.

### Indemnities

The study carries very minimal risk to the study participants, which is why we have no compensation or insurance plan for the participants. However, we will provide US$5 per home visit to FCHVs for transportation expenses.

### Publication plan

We are planning to publish the results of the trial as a peer-reviewed publication in a PubMed-listed journal.

## Discussion

Models of health service provided by lay health workers are operational in several underresourced countries, including India, Kenya, Uganda, Ghana, Ethiopia, South Africa, and China [30]. Previous studies have shown that community health worker programs for health care delivery are significantly beneficial in maternal and child health as well as infectious disease control [30, 31]. However, involving community health workers for noncommunicable diseases has not gained much attention until recently. This study pioneers the investigation of a community-based intervention to reduce blood pressure in resource-poor countries. If our study shows that the intervention is effective, a scaled-up approach could produce an important reduction in cardiovascular disease burden due to hypertension. The proposed study will increase knowledge on how to control blood pressure by mobilizing community health volunteers in Nepal. Thus, the research output of this study has the potential to bring immediate benefits to address noncommunicable diseases in Nepal. Assessing the FCHVs’ capacity for identifying hypertensive patients and for reducing risk factors related to hypertension may contribute to developing a policy that can be scaled up to a national level. The lessons learned from this study may also be replicated in other rural areas of Nepal and elsewhere in the world with similar settings.

## Trial status

Trial has been closed to participant accrual, but trial is ongoing. The endpoint assessment of all the participants will be completed by the end of November 2016. The final outcome assessment of all the participants will be completed by the end of December 2016. The schedule of enrollment, interventions, and assessments is presented in Additional file 1.

## Abbreviations

DBP, diastolic blood pressure; FCHV, female community health volunteer; SBP, systolic blood pressure; WHO, World Health Organization

## Additional file

Additional file 1: Schedule of enrollment, interventions, and assessments. (DOC 46 kb)
