# Supplementary material for: Community-based intervention for blood pressure reduction in Nepal (COBIN trial): study protocol for a cluster-randomized controlled trial
Source: Trials. 2016 Jun 18;17:292. doi: 10.1186/s13063-016-1412-3 (PMC4912718; doi:10.1186/s13063-016-1412-3)
Supplement: Additional file 1: — Schedule of enrollment, interventions, and assessments. (DOC 46 kb) [file 13063_2016_1412_MOESM1_ESM.doc]

Table 3: Schedule of enrolment, interventions and assessments (SPIRIT Figure)

|  | **STUDY PERIOD** | | | | | | |
| --- | --- | --- | --- | --- | --- | --- | --- |
|  | **Enrolment** | **Allocation** | **Post-allocation** | | | | **Close-out** |
| **TIMEPOINT**  **(Months)** | ***6*** | **1** | ***1*** | ***4*** | ***4*** | ***4*** | ***3*** |
| **ENROLMENT:** |  |  |  |  |  |  |  |
| **Eligibility screen** | X |  |  |  |  |  |  |
| **Informed consent** | X |  |  |  |  |  |  |
| ***Informed consent with FCHVs*** | X |  |  |  |  |  |  |
| **Allocation** |  | X |  |  |  |  |  |
| **INTERVENTIONS:** |  |  |  |  |  |  |  |
| ***[Training of FCHVs]*** |  |  | X |  |  |  |  |
| ***[Home visit by FCHVs]*** |  |  |  | X | X | X |  |
|  |  |  |  |  |  |  |  |
| **ASSESSMENTS:** |  |  |  |  |  |  |  |
| ***Baseline variable***  ***Socio-****Demographic measurement (age, sex, ethnicity, monthly income), behavioural measurement (tobacco use, alcohol consumption, diet, physical activity, salt intake)*  *Physical measurement (Height, weight, waist circumference, hip circumference, pulse)* | X |  |  |  |  |  | x |
| ***Outcome variable***  *SBP, DBP* | x |  |  |  |  |  | X |

.
